# Portal Hypertension, Variceal Bleeding, and High Output Cardiac Failure Secondary to an Intrahepatic Arterioportal Fistula

**DOI:** 10.1155/1993/95416

**Published:** 1993

**Authors:** Andrew J. Oishi, David M. Nagorney, Kenneth J. Cherry

**Affiliations:** 1 Department of General Surgery Mayo Graduate School of Medicine Rochester Minnesota USA; 2 Department of General Surgery Mayo Clinic 200 First Street, SW Rochester MN 55905 USA; 3 Division of Vascular Surgery Mayo Clinic Rochester Minnesota USA

## Abstract

Intrahepatic arterioportal fistulas (APF) are uncommon complications following hepatic trauma. Large fistulas can result in portal hypertension and cardiovascular compromise. A 46-year-old patient is described who presented with portal hypertension, variceal bleeding, and high output cardiac failure due to a large intrahepatic APF. Surgical closure of the APF by hepatic resection successfully resolved the portal hypertension, prevented further variceal hemorrhage, and restored normal cardiovascular function.


*HPB Surgery*, 1993, Vol. 7, pp. 53–59
Reprints available directly from the publisher
Photocopying permitted by license only

© 1993 Harwood Academic Publishers GmbH
Printed in the United States of America

### CASE REPORT

### PORTAL HYPERTENSION, VARICEAL BLEEDING, AND HIGH OUTPUT CARDIAC FAILURE SECONDARY TO AN INTRAHEPATIC ARTERIOPORTAL FISTULA

ANDREW J. OISHI
*Resident, Department of General Surgery, Mayo Graduate School of Medicine, Rochester, Minnesota, USA*

DAVID M. NAGORNEY
*Associate Professor of Surgery, Consultant, Department of General Surgery, Mayo Clinic, Rochester, Minnesota, USA*

KENNETH J. CHERRY
*Associate Professor of Surgery, Consultant and Chairman, Division of Vascular Surgery, Mayo Clinic, Rochester, Minnesota, USA*

Intrahepatic arterioportal fistulas (APF) are uncommon complications following hepatic trauma. Large fistulas can result in portal hypertension and cardiovascular compromise. A 46-year-old patient is described who presented with portal hypertension, variceal bleeding, and high output cardiac failure due to a large intrahepatic APF. Surgical closure of the APF by hepatic resection successfully resolved the portal hypertension, prevented further variceal hemorrhage, and restored normal cardiovascular function.

KEY WORDS: Arterioportal fistula, portal hypertension, hepatic resection

### INTRODUCTION

An intrahepatic arterioportal fistula (APF) is an uncommon complication following blunt abdominal trauma with hepatic injury. Small fistulas usually are clinically insignificant and can close spontaneously[1]. However, some fistulas persist and can lead to pathologic changes in hepatic hemodynamics. Chronic arterial inflow into the portal vein lead to portal venous hypertension and esophageal varices with bleeding. Associated morbidity and mortality can be considerable. Timely diagnosis is essential and these cases are often challenging management problems. We present such a case for review.

---

Address correspondence to: David M. Nagorney, M.D., Department of Surgery, Mayo Clinic, 200 First Street, SW, Rochester, MN 55905, USA. Tel.(507) 284-2644

### CASE REPORT

A 46-year-old caucasian male was involved in a high speed motor vehicle accident in 1959. He sustained blunt abdominal trauma and required multiple transfusions of whole blood for stabilization. An emergency abdominal exploration revealed a large stellate laceration in the right lobe of the liver and partial right hepatic lobectomy was performed. He subsequently recovered without incident. He remained well until February 1989 when an episode of hematemesis and orthostatic hypotension prompted transfusion resuscitation at a local hospital.

Esophagogastroduodenoscopy revealed large nonbleeding esophageal and gastric varices. No other source of bleeding was identified. Abdominal computed tomography showed marked dilatation of the portal vein and splenomegaly. A hepatic angiogram demonstrated a large APF in the right hepatic lobe with retrograde flow of contrast through the portal venous system. He was treated conservatively and discharged from the hospital.

Two and a half weeks later, in March 1989, the patient had a second episode of hematemesis and hypotension requiring readmission and blood transfusions. He also developed signs of progressing liver failure with ascites. Repeat upper endoscopy was performed which again showed large esophageal and gastric varices with stigmata of recent hemorrhage. The patient was transferred to the Mayo Clinic for further evaluation and treatment. Vital signs on admission included blood pressure of 130/88 mm Hg and pulse of 92. There were no orthostatic symptoms. He was not jaundiced. His abdomen was non-distended, soft, and nontender. His liver and spleen were not palpable. A loud continuous bruit was heard in his right upper quadrant on auscultation. Physical examination was otherwise unremarkable.

Laboratory studies revealed a hemoglobin—10.4 gm/dL, aspartase aminotransferase (AST)—66 U/L, alkaline phosphatase—371 U/L, total bilirubin—1.6 mg/dL, prothrombin time—12.2 seconds, and activated partial thromboplastin time—32.2 seconds. A chest X-ray showed cardiomegaly. An abdominal ultrasound demonstrated extensive tortuous collateral vessels with turbulent blood flow adjacent to the right hepatic lobe with extension into the retroperitoneum. Visceral angiography showed a large aneurysmal common hepatic artery with early filling of the portal venous system (Figure 1). Measurement of hepatic vein pressures showed a free hepatic vein pressure of 18 mm Hg with a wedge of 21 mm Hg. A diagnosis of APF causing portal hypertension with esophageal hemorrhage and compensated high output cardiac failure was made. Surgical division of the APF was undertaken to decompress the portal system and to restore normal hemodynamics.

At laparotomy, a moderate amount of ascitic fluid was encountered. Abdominal exploration revealed mild splenomegaly with varices along the lesser and greater curves of the stomach and retroperitoneum. A large aneurysmal common hepatic artery was identified (Figure 2). There was an obvious palpable thrill within the right hepatic lobe. No extrahepatic arterial-portal venous communication was identified. Hepatic arterial anatomy was classic with both the left and right hepatic arteries arising from the common hepatic artery. The common hepatic artery aneurysm was resected to its origin from the celiac axis. A right hepatic lobectomy was performed. A gastroduodenal artery to left hepatic artery anastomosis was constructed to insure arterial blood supply to the left lobe of the liver. After

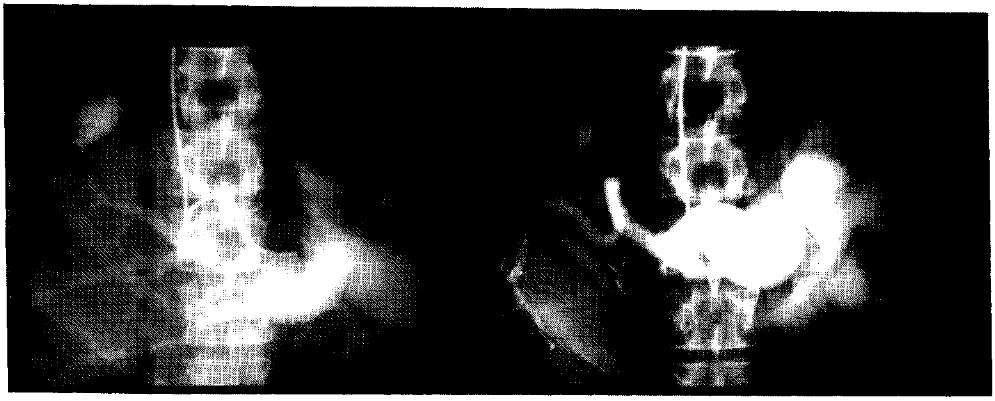

**Figure 1** (left) Preoperative celiac angiogram demonstrating ectatic aneurysmal common hepatic artery and early filling of a dilated portal vein. (right) Delayed film showing complete filling of the dilated left and right branches of the portal vein.

**Figure 2** Intraoperative photograph of the tortuous, common hepatic artery aneurysm. *(See colour plate I at back of issue).*

completion of the right lobectomy, intraoperative cardiac output fell from 12.5 L/min to 7.2 L/min and portal vein pressure decreased from 33 to 15 mm Hg. His postoperative course was uneventful. Magnetic resonance imaging demonstrated patency of both the gastroduodenal hepatic artery bypass and the portal vein. Duplex ultrasound confirmed hepatopedal portal vein blood flow. He has remained

**Figure 3** (top) Gross pathologic specimen of right hepatic lobe with probe through the hepatic artery-portal vein fistula. (bottom) Close-up photograph of arterioportal fistula. *(See colour plate II at back of issue).*

well without recurrent variceal hemorrhage or cardiac compromise since his dismissal from the hospital.

### DISCUSSION

Although posttraumatic APF is being reported with increasing frequency, we feel the management of this case was unique for two reasons. First, in addition to a right hepatic lobectomy and resection of the hepatic artery aneurysm, a gastroduodenal artery to left hepatic artery bypass was constructed to maintain hepatic arterial blood flow to the remaining lobe of the liver. This reconstruction resulted in minimal hepatic dysfunction postoperatively and contributed to his rapid recovery. Secondly, a marked decrease in both portal vein pressure and cardiac output was demonstrated immediately following closure of the APF, illustrating the reversibility of the pathologic changes of APF.

Since the first reported case by Sachs in 1892,[2] APF has been a recognized cause of portal hypertension and variceral hemorrhage. However, an attempted surgical closure of APF was not reported until 1954 by Madding *et al.*[3] They sequentially ligated branches of the celiac axis until the fistula thrill could no longer be palpated. Despite this approach, the patient died of continued hemorrhage from esophageal varices. The first successful surgical repair of APF was reported by Wheeler and Warren in 1957[4]. They described a patient with bleeding varices in whom a diagnosis of APF was made only after three abdominal procedures. Following a distal splenorenal shunt, the patient developed a loud epigastric murmur that led to the diagnosis. Ligation of branches of the hepatic artery and obliteration of a hepatic artery aneurysm at a fourth operation lead to a successful outcome.

Trauma and neoplasm are the most common causes of APF[5,6]. Iatrogenic injury from percutaneous needle biopsy and cholangiography are the most frequent traumatic causes. Often these AFP's are small and close spontaneously[5-7]. Blunt and penetrating trauma are responsible for the more clinically significant APF's which require intervention[7,8]. Reported intervals between hepatic injury and diagnosis of APF have ranged from a few hours to 39 years[5,9].

Although some patients are asymptomatic, most present with a range of symptoms. Mild to moderate abdominal pain and diarrhea secondary to congestive vascular enteropathy may be early findings. Signs of more advanced disease include portal hypertension with bleeding varices and ascites or other sign of liver failure. Hemobilia has also been reported[9]. The patient described here presented with high output cardiac failure and associated cardiomegaly which resolved postoperatively. Although uncommon with APF due to the sinusoidal nature of the hepatic parenchyma which diminishes the velocity of blood flow through the fistula, this finding has been reported[8,11-14].

Diagnosis is usually difficult. Past history and physical examination may be helpful. An epigastric bruit in a patient with a history of unexplained liver failure or portal hypertension is particularly suggestive. Current hepatobiliary imaging, whether noninvasive or invasive, can confirm clinical suspicions. Computed tomography (CT), particular the rapid-sequence CT scan, has proven especially useful[15]. Early opacification of the portal vein after injection of intravenous contrast is a reliable finding of APF. In addition, a difference in attenuation between the right and left hepatic lobes can be a clue to the location of an intrahepatic fistula. Late findings that can be seen on CT scan include dilatation of the portal vein, splenomegaly, and retroperitoneal venous collateral vessels. Doppler ultrasonography (US) is another useful noninvasive test. US can demonstrate aneurysmal dilatation of visceral arteries and retrograde, pulsatile portal venous flow.

Moreover, US can demonstrate the presence of collateral venous circulation[16,17]. Magnetic resonance imaging (MRI) can also be helpful in delineating intra-abdominal vessels. MRI can demonstrate flow in both the hepatic artery and portal vein but generally has not proven superior to the combination of CT and US in its ability to image APF. The gold standard for preoperative diagnosis of APF remains visceral arteriography. Angiography can accurately demonstrate both the site of the fistula and the presence of any associated aneurysmal disease, although aneurysm size is better determined by CT or MRI.

The management of posttraumatic fistulas is determined by multiple factors: size and location of the fistula, presenting symptoms, duration between injury and diagnosis, patient performance status, and past and concurrent hepatic disease. Small fistulas which are located extrahepatically in an asymptomatic patient can often close spontaneously and may warrant a trial of observation[1,12,18]. Larger fistulas and those presenting after remote hepatic injury and associated with significant symptoms require intervention. Angiographic embolization of the fistula can be a simple and effective means of cure providing that the fistula is not too large and is accessible. Results with embolization have been very good. Closure by embolization with Gelfoam, steel coils, detachable balloons, or bucrylate has been achieved in 92% to 100% of patients when attempted. Nearly all patients have had complete relief of symptoms in these selected situations after embolization, and APF has recurred in less than 20% of patients[8,17,19-21]. Surgical intervention is reserved for those patients who have fistulas too large for safe embolization[19,21]. A range of operative procedures have been described. Simple ligation of feeding arterial branches with or without division of the fistula has been used selectively. However, intrahepatic location and the presence of multiple arterial collateral vessels to the AFP often make this procedure ineffective. Hepatic resection of liver parenchyma containing the AFP and ligation of either a feeding branch of the hepatic artery or the common hepatic artery has proven definitive when employed[9,10,22-23]. Arterial flow to the contralateral hepatic lobe should be maintained to insure optimal liver function. Although prolonged periods of arterial inflow may be associated histologically with portal venous sclerosis, persistent portal hypertension after resection of APF has not been recognized clinically.

### <B>References

1. Tanaka, H., Iwai, A., Sugimota, H., *et al.* (1991) Intrahepatic arterioportal fistula after blunt hepatic trauma: Case reports. *J.Trauma*, **31**(1), 143–146
2. Sachs, R. (1892) Zur casuisitik der gefasserkrankungen. *Deutsche Medianische Wochenschr*, **18**, 443–447
3. Madding, G.F., Smith, W.L. and Hershberger, L.R. (1954) Hepatoportal arteriovenous fistula. *JAMA*, **156**(96), 593–596
4. Wheeler, H.B. and Warren, R. (1957) Duodenal varices due to portal hypertension from arteriovenous aneurysm: Report of a case. *Ann.Surg.*, **146z**(2), 229–238
5. Strodel, W.E., Eckhauser, F.E., Lemmer, J.H. *et al.* (1987) Presentation and perioperative management of arterioportal fistulas. *Arch.Surg.*, **122**, 563–571
6. Missavage, A.E., Jones, A.M., Walt, A.J. *et al.* (1984) Traumatic arteriovenous fistula causing portal hypertension and variceal bleeding. *J.Trauma*, **24**(4), 355–358
7. Okuda, K., Musha, H. K+Nakajima, Y. *et al.* (1978) Frequency of intrahepatic arteriovenous fistula as a sequela to percutaneous needle puncture of the liver. *Gastroenterology*, **74**(6), 1204–1207
8. Kaude, J., Dudgeon, D.L. and Talbert, J.L. (1969) The role of selective angiography in the diagnosis and treatment of hepatoportal arteriovenous fistula. *Radiology*, **92**, 1271–1272
9. Malette, W.G., Griffen, W.O., Stivers, J.R. *et al.* (1973) Traumatic intrahepatic fistula of the hepatic artery and portal vein. *Am.Surg.*, **39**, 164–169

10. Cleveland, R.J., Jackson, B.M., Newman, P.H. *et al.* (1970) Traumatic artery-portal vein fistula with associated hemobilia. *Ann.Surg.*, **171**(3), 451–451
11. Foster, J.H. and Sandblom, P. (1961) Portal hypertension secondary to a hepatoportal arteriovenous fistula. *Ann.Surg.*, **154**(2), 300–304
12. Foley, W.J., Turcotte, J.G., Hoskins, P.A. et al. (1971) Intrahepatic arteriovenous fistulas between the hepatic artery and portal vein. *Ann.Surg.*, **174**(5), 849–855
13. Cohen, A. and DeBakey, M.E. (1960) Successful repair of traumatic arteriovenous fistulas between hepatic artery and portal vein and right renal artery and inferior vena cava. *Surgery*, **48**(3), 548–553
14. Schilling, J.A., McKee, F.W. and Wilt, W. (1950) Experimental hepatic-portal arteriovenous anastomosis. *Surg.Gynecol.Obstet.*, **90**, 473–480
15. Takayasu, K., Moriyama, N., Ishikawa, T. *et al.* (1985) Large spontaneous intrahepatic arterioportal fistula demonstrated by rapid sequential computed tomographic scan: Report of two cases. *Gastroenterology*, **88**, 564–570
16. van Leeuwen, M.S. (1990) Doppler ultrasound in the evaluation of portal hypertension. *Clin.Diagn.Ultrasound*, **26**, 53–76
17. Tarazov, P.G. and Prozorovskij, K.V. (1991) Intrahepatic spontaneous arterioportal fistula: Duplex ultrasound diagnosis and angiographic treatment. *Am.J.Gastroenterology*, **86**(6), 775–778
18. Schmidt, B., Bhatt, G.M. and Abo, M.N. (1980) Management of post-traumatic vascular malformations of the liver by catheter embolization. *Am.J.Surg.*, **140**, 332–335
19. Mcneese, S., Finck, E. and Yellin, A.E. (1980) Definitive treatment of selected vascular injuries and post-traumatic arteriovenous fistulas by angiographic embolization. *Am.J.Surg.*, **140**, 252–259
20. Van Haeften, F.F. and Broker, F.H. (1984) Post-traumatic intrahepatic arteriovenous fistula. *Injury*, **15**(5), 311–315
21. Redmond, P.L. and Kumpe, D.A. (1988) Embolization of an intrahepatic arterioportal fistula: Case report and review of the literature. *Cardiovasc.Interven.Rad.*, **11**, 274–277
22. Shumacker, H.B. and Waldhausen, J.A. (1961) Intrahepatic arteriovenous fistula of hepatic artery and portal vein. *Surg.Gynecol.Obstet.*, **112**, 497–501
23. Olsen, W.R. (1982) Late complications of central liver injuries. *Surgery*, **92**(4), 773–743
24. Strickler, J.H., Lufkin, N. and Rice, C. (1952) Hepatic portal arteriovenous fistula: A case report. *Surgery*, **31**(4), 583–589

*(Accepted by S. Bengmark 21 January 1993)*

